# Differential diagnostic value of total alpha-synuclein assay in the cerebrospinal fluid between Alzheimer’s disease and dementia with Lewy bodies from the prodromal stage

**DOI:** 10.1186/s13195-020-00684-5

**Published:** 2020-09-29

**Authors:** Olivier Bousiges, Nathalie Philippi, Thomas Lavaux, Armand Perret-Liaudet, Ingolf Lachmann, Caroline Schaeffer-Agalède, Pierre Anthony, Anne Botzung, Lucie Rauch, Barbara Jung, Paulo Loureiro de Sousa, Catherine Demuynck, Catherine Martin-Hunyadi, Benjamin Cretin, Frédéric Blanc

**Affiliations:** 1 Laboratory of Biochemistry and Molecular Biology, University Hospital of Strasbourg, 67000 Strasbourg, France; 2 Laboratoire de Neurosciences Cognitives et Adaptatives (LNCA), University of Strasbourg, 67000 Strasbourg, France; 3 CNRS UMR7364, 67000 Strasbourg, France; 4 CM2R (Research and Resources Memory Centre), Geriatric Day Hospital and Neuropsychology Unit, Geriatrics Department, University Hospitals of Strasbourg, Strasbourg, France; 5 University of Strasbourg and CNRS, ICube Laboratory UMR 7357 and FMTS (Fédération de Médecine Translationnelle de Strasbourg), Team IMIS, Strasbourg, France; 6 Neurochemistry Laboratory, Biochemistry Department, Centre de Biologie et Pathologie Est, Hospices Civils de Lyon, 59 bd Pinel, 69677 Bron, France; 7 BIORAN Team, Lyon Neuroscience Research Center, CNRS UMR 5292 - INSERM U1028, Université de Lyon - Université Claude Bernard, 95 bd Pinel, 69675 Bron, France; 8 Center for Memory Resources and Research, Hospices Civils de Lyon, Charpennes Hospital, Lyon 1 University, 69100 Villeurbanne, France; 9 AJ Roboscreen GmbH, Hohmannstraße 7, 04129 Leipzig, Germany; 10 Geriatrics Department, General Hospital Centre, CM2R, Geriatric Day Hospital, Colmar, France

**Keywords:** Dementia with Lewy bodies, Alzheimer’s disease, Prodromal, Dementia, Cerebrospinal fluid biomarkers, Total alpha-synuclein

## Abstract

**Background:**

Several studies have investigated the value of alpha-synuclein assay in the cerebrospinal fluid (CSF) of Alzheimer’s disease (AD) and dementia with Lewy bodies (DLB) patients in the differential diagnosis of these two pathologies. However, very few studies have focused on this assay in AD and DLB patients at the MCI stage.

**Methods:**

All patients were enrolled under a hospital clinical research protocol from the tertiary Memory Clinic (CM2R) of Alsace, France, by an experienced team of clinicians. A total of 166 patients were included in this study: 21 control subjects (CS), 51 patients with DLB at the prodromal stage (pro-DLB), 16 patients with DLB at the demented stage (DLB-d), 33 AD patients at the prodromal stage (pro-AD), 32 AD patients at the demented stage (AD-d), and 13 patients with mixed pathology (AD+DLB). CSF levels of total alpha-synuclein were assessed using a commercial enzyme-linked immunosorbent assay (ELISA) for alpha-synuclein (AJ Roboscreen). Alzheimer’s biomarkers (t-Tau, P-Tau, Aβ42, and Aβ40) were also measured.

**Results:**

The alpha-synuclein assays showed a significant difference between the AD and DLB groups. Total alpha-synuclein levels were significantly higher in AD patients than in DLB patients. However, the ROC curves show a moderate discriminating power between AD and DLB (AUC = 0.78) which does not improve the discriminating power of the combination of Alzheimer biomarkers (AUC = 0.95 with or without alpha-synuclein). Interestingly, the levels appeared to be altered from the prodromal stage in both AD and DLB.

**Conclusions:**

The modification of total alpha-synuclein levels in the CSF of patients occurs early, from the prodromal stage. The adding of alpha-synuclein total to the combination of Alzheimer’s biomarker does not improve the differential diagnosis between AD and DLB.

**Trial registration:**

ClinicalTrials.gov, NCT01876459 (AlphaLewyMa)

## Background

Dementia with Lewy bodies (DLB) is the most frequent dementia after Alzheimer’s disease (AD). The clinical diagnosis of DLB is well defined and regularly revised [1–4]. Despite the prevalence of DLB, only one third of patients are correctly diagnosed, leaving two thirds of these patients undiagnosed or misdiagnosed [5]. DLB is complicated to diagnose due to its similarity to AD and Parkinson’s disease (PD). DLB is close to AD because of cognitive decline (episodic memory, working memory, executive functions) and to PD because of parkinsonism and for the pathophysiological aspect because of the alpha-synuclein (α-syn) aggregation. What happens first in DLB is the cognitive decline, which explains the frequent misdiagnosis with AD. Furthermore, the cognitive and motor symptoms found in DLB can be found in other diseases, which makes differential diagnosis complex. Like other neurodegenerative diseases, DLB progresses insidiously and slowly to a demented state. We now know the importance of early treatment in neurodegenerative disease. Consequently, when effective treatment arrives on the market, we will need to be able to treat patients at a prodromal stage. It is therefore important to be able to diagnose these patients early.

The prodromal stage of DLB (pro-DLB), also called mild cognitive impairment due to Lewy bodies (MCI-LB), has recently been described in detail: the first criteria of this prodromal stage are similar to the stage of dementia with the difference that decrease in functional capacity is either non-existent or minimal [6].

It is challenging to diagnose DLB at an early stage and, if we add to this the neurological comorbidities that are common in the elderly and more particularly with DLB [7], it is easy to understand the difficulty in diagnosing this type of disease. For all these reasons, it is clear that specific biomarkers need to be found to allow the differential diagnosis of DLB.

To date, many studies have focused on biomarkers used in clinical routine, i.e., Alzheimer’s biomarkers (t-Tau, P-Tau, Aβ42, Aβ40; for a review, see [8]). These studies have shown the great interest of these biomarkers, especially t-Tau, P-Tau, and the ratio Aβ42/Aβ40, in the differential diagnosis between AD and DLB, especially at the prodromal stage, where the differential diagnosis is even more delicate [9, 10].

DLB and PD, as well as multiple system atrophy (MSA), have one thing in common, namely the α-syn aggregation leading to Lewy body formation. That is why these pathologies are part of a group of disorders known as synucleinopathies. Based on these aggregative phenomena and on the way in which amyloid and Tau biomarkers are used in AD, these α-syn-related proteins could be of interest in the differential diagnosis of DLB. Studies that have included the measurement of total α-syn are relatively numerous and not always consensual.

The aim of our study was therefore to determine the discriminating ability of the α-syn assay in cerebrospinal fluid (CSF), without or in combination with the standard AD-related biomarkers, between DLB and AD patients, in both demented and mild cognitive impairment (MCI) patients.

## Methods

### Patients

All patients were enrolled under a hospital clinical research protocol called AlphaLewyMA (registered in ClinicalTrials.gov: https://clinicaltrials.gov/ct2/show/NCT01876459) from the tertiary Memory Clinic (CM2R) of Alsace by an experienced team of neurologists, geriatricians, and neuropsychologists between June 2013 and June 2018. The CM2R of Alsace comprises 3 different centers, two at the University Hospitals of Strasbourg (*CHU Hautepierre* and *Hôpital de la Robertsau*) and one at *Hôpitaux Civils de Colmar*. Patients underwent detailed clinical evaluation, a large neuropsychological evaluation, blood examination, brain MRI (3 Tesla), and lumbar puncture for CSF biomarkers as previously described [11].

DLB patients were selected according to McKeith’s criteria (probable DLB, based on the existence of two core symptoms in addition to cognitive decline) for DLB demented (DLB-d) and prodromal DLB (pro-DLB) patients also called mild cognitive impairment with Lewy bodies (MCI-LB) [3, 6]. To note, Parkinsonism is present in 81.6% of the pro-DLB patients. However, Parkinsonism is in any case very subtle. For information, fluctuations were assessed with the Mayo Clinic Fluctuations Scale [12]. The Hallucinations Parkinson’s disease-associated psychotic symptoms questionnaire was used to evaluate the presence of hallucinations [13]. RBD was evaluated using a questionnaire based on the article by Gjerstad et al. [14], simplified into two questions for the patient and the caregiver, one concerning movements during sleep and the other concerning vivid dreams and nightmares.

Patients with AD were selected according to Albert’s criteria [15] and Dubois’ criteria [16] for patients with pro-AD and McKhann’s criteria [17] and Dubois’ criteria [16] for demented AD patients.

Patients were considered to have DLB and AD when they meet both the Dubois’ criteria and the McKeith’s criteria concurrently. For example, a patient with memory storage disorders, a CSF in favor of AD, and two of the four clinical criteria for DLB was considered to have both DLB and AD.

**Fig. 1:**
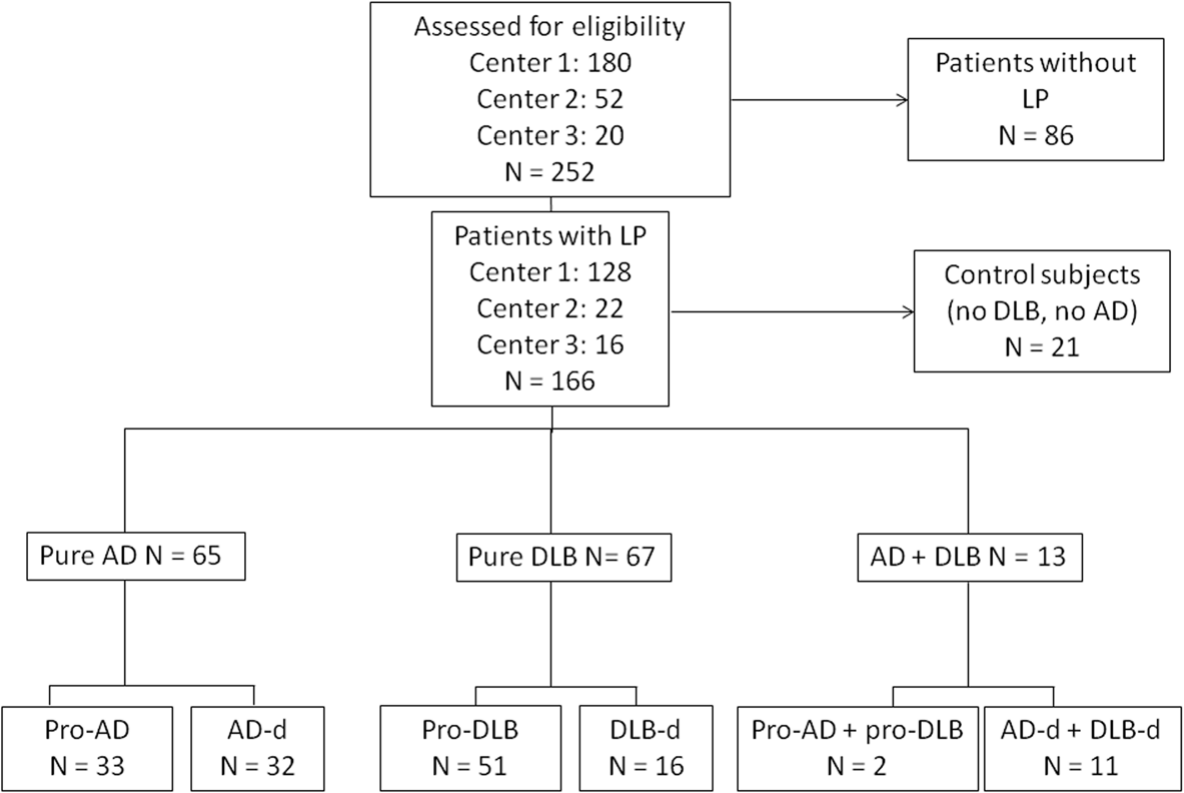
Flowchart of patient selection from AlphaLewyMA study. Center 1: CHU de Hautepierre; center 2: Hôpital de la Robertsau; center 3: Hôpitaux Civils de Colmar; AD, Alzheimer’s disease; DLB, dementia with Lewy bodies; Pro-AD, prodromal AD; Pro-DLB, prodromal DLB; AD-d, AD demented; DLB-d, DLB demented

Table 1 summarizes the main clinical information of the patients at the time of lumbar puncture. A total of 166 patients were included in this study: 21 control subjects (CS group), 51 patients with DLB at the prodromal stage (pro-DLB group), 16 patients with DLB at the demented stage (DLB-d group), 33 AD patients at the prodromal stage (pro-AD group), 32 AD patients at the demented stage (AD-d group), and 13 patients with both the criteria of AD and criteria of probable DLB [3], divided into two groups (pro-AD/DLB group [*n* = 2] and AD/DLB-d group [*n* = 11]); data of the latter two groups were analyzed separately from the data of patients with pure AD or pure DLB (see flowchart in Fig. 1). The CS group consisted of patients originally included in the study with cognitive disorders as found in AD and DLB, who, after follow-up in the study, were found to have neither AD nor DLB. The CS group had various diagnoses, defined according to international criteria (for details, see Table 1).

**Table 1:** Clinical and demographic characteristics of patient groups and their biomarker values

				**DLB** ***N*** **= 67**		**AD** ***N*** **= 65**		**AD+DLB** ***N*** **= 13**					
				**Pro-DLB** ***N*** **= 51**	**DLB-d** ***N*** **= 16**	**Pro-AD** ***N*** **= 33**	**AD-d** ***N*** **= 32**	**Pro-AD/DLB** ***N*** **= 2**	**AD/DLB-d** ***N*** **= 11**	**CS**^**f**^ ***N*** **= 21**		**Test statistic,** ***P***	**Post hoc**^**g**^
**Age, years**^**a**^				66.2 (9.0)	75.4 (7.0)	71.1 (8.0)	70.8 (8.2)	77.5 (4.9)	77.3 (6.3)	68.5 (9.0)		*H* = 19.88, *P* = .0013	Pro-DLB < DLB-d and AD/DLB-d *P* < 0.05
**Gender (F/M)**				27/24	12/4	15/18	20/12	1/1	6/5	11/10		*χ*^2^ = 4.677, *P* = .4566	
**MMSE score**^**b**^				27.3 (2.4)	21.0 (4.0) (1ND)	26.4 (2.7)	21.4 (4.3)	26.5 (2.1)	20.7 (3.4)	26.9 (2.3)		*H* = 80.34, *P* < .0001	Pro and CS > d
**Hallucinations**^**c, i**^				68.6%	56.3%	12.5% (1ND)	32.3% (1ND)	50%	63.6%	33.3%		*χ*^2^ = 29.99 *P* < .0001	
**Fluctuations**^**c, j**^				83.3% (3ND)	75.0%	9.7% (2ND)	38.7% (1ND)	100%	90.9%	38.1%		*χ*^2^ = 55.04, *P* < .0001	
**Parkinsonism**			**Rigidity** **0/1/2/3/4**	24/29/1/0/0 (2ND)	3/7/5/1/0	27/5/1/0/0	23/8/1/0/0	0/2/0/0/0	2/9/0/0/0	11/8/0/2/0		*H* = 37.99, *P* < .0001	pro-DLB > pro-AD and AD-d; DLB-d > pro-AD and AD-d; AD/DLB-d > pro-AD
			**Akinesia** **0/1/2/3/4**	22/22/6/0/0 (2ND)	3/8/3/2/0	29/4/0/0/0	27/4/1/0/0	0/2/0/0/0	2/7/1/1/0	17/2/2/0/0		*H* = 46.15, *P* < .0001	CS < DLB-d and AD/DLB-d; pro-DLB > pro-AD and AD-d; DLB-d and AD/DLB-d > pro-AD and AD-d
			**Tremor at rest** **0/1/2/3/4**	32/15/1/0/0 (3ND)	11/5/0/0/0	30/3/0/0/0	30/1/0/0/0 (1ND)	1/1/0/0/0	9/2/0/0/0	19/2/0/0/0		*H* = 16.87, *P* < .0048	Pro-DLB > AD-d
**RBD**^**c**, k^				43.8% (3ND)	43.8%	6.1%	19.4% (1ND)	0%	27.3%	33.3%		*χ*^2^ = 16.99, *P* = .00045	
**Hippocampi atrophy**^**d**^ **0/1/2/3/4**			**Left hippocampus**	23/15/6/5/1 (1ND)	1/4/7/1/3	3/19/8/2/0 (1ND)	5/9/12/2/1 (3ND)	1/0/0/1/0	0/6/4/0/1	10/4/4/2/0 (1ND)		*H* = 21.00, *P* = .0008	DLB-d > CS and pro-DLB
			**Right hippocampus**	23/17/8/1/1 (1ND)	2/6/3/1/4	5/18/8/1/0 (1ND)	7/12/8/1/1 (3ND)	1/0/0/1/0	0/6/4/0/1		8/6/6/0/0 (1ND)	*H* = 16.68, *P* = .0051	
**FCSRT**^**e**^				22% (1ND)	71.4% (2ND)	78.1% (1ND)	93.5% (1ND)	50%	100% (1ND)	30.0% (1ND)		*χ*^2^ = 62.7, *P* < .0001	
**CSF biomarkers**^**h**^			**t-Tau (ng/L)**	271 [108]	306 [108]	630 [339]	628 [231]	582 [486]	627 [307]	265 [93]		*H* = 88.14 *P* < .0001	CS, pro-DLB, DLB-d < pro-AD, AD-d, AD+DLB-d
			**P-Tau (ng/L)**	43 [15]	47 [14]	91 [33]	81 [22]	76 [58]	92 [44]	43 [17]		*H* = 90.34 *P* < .0001	CS, pro-DLB, DLB-d < pro-AD, AD-d, AD+DLB-d
			**Aβ42 (ng/L)**	911 [292]	742 [268]	642 [299]	518 [571]	688 [194]	437 [181]	1002 [256]		*H* = 59.30 *P* < .0001	CS, pro-DLB > pro-AD, AD-d, AD+DLB-d
			**t-α-synuclein**	118 [49]	112 [62]	197 [77]	183 [114]	145 [29]	187 [86]	141 [57]		*H* = 35.55 *P* < .0001	Pro-DLB < pro-AD and AD-d; DLB-d < pro-AD
			**Aβ40 assays**	**DLB** ***N*** **= 34**		**AD** ***N*** **= 25**		**AD+DLB** ***N*** **= 6**					
				**Pro-DLB** ***N*** **= 28**	**DLB-d** ***N*** **= 6**	**Pro-AD** ***N*** **= 16**	**AD-d** ***N*** **= 9**	**Pro-AD/DLB** ***N*** **= 1**	**AD/DLB-d** ***N*** **= 5**	**CS**^**f**^ ***N*** **= 11**			
			**Aβ40 (ng/L)**	9081 [2320]	8303 [2681]	13,892 [6575]	10,293 [3891]	22,700	12,423 [4468]	11,308 [4825]		*H* = 10.08 *P* = 0.0731	
			**Aβ42/Aβ40**	0.107 [0.035]	0.107 [0.048]	0.052 [0.021]	0.051 [0.022]	0.036	0.039 [0.005]	0.102 [0.029]		*H* = 42.13 *P* < .0001	CS and pro-DLB > pro-AD, AD-d, AD+DLB-d; DLB-d > AD+DLB-d

*CDR* clinical dementia rating, *MMSE* Mini-Mental Status Examination, *N* number, *RBD* rapid eye movement sleep behavior disorder, *FCSRT* Free and Cued Selective Reminding Test

^a^Age at time of lumbar puncture and cognitive evaluation. Mean (standard deviation)

^b^Mean (standard deviation)

^c^Percentage

^d^According to [18]

^e^Percentage of deficient patients

^f^The group included patients suffering from depression (*n* = 1); neurosis (*n* = 1); vascular dementia and depression (*n* = 1), sleep apnea syndrome and primary age-related tauopathy (PART) (*n* = 1), vascular MCI and sleep apnea syndrome (*n* = 1), traumatic brain injury and left parietal meningeal hemorrhage (*n* = 1), corticobasal degeneration (CBD) (*n* = 1); Gougerot-Sjögren’s syndrome (*n* = 1); fronto-insular low-grade glioma (*n* = 1); cognitive impairment due to diabetes (*n* = 1); temporo-insular cavernoma (*n* = 1); vascular dementia and frontotemporal dementia (FTD) (*n* = 1); temporal epilepsy (*n* = 2); progressive supranuclear palsy (PSP) (*n* = 3); vascular dementia (*n* = 1); primary age-related tauopathy (PART) (*n* = 2); and stroke (*n* = 1)

^g^Kruskal-Wallis post hoc test (H)

^h^CSF biomarkers at time of cognitive evaluation. Mean [standard deviation]

^i^The Hallucinations Parkinson’s disease-associated psychotic symptoms questionnaire was used to evaluate the presence of hallucinations [48]

^j^Fluctuations were assessed with the Mayo Clinic Fluctuations Scale [49]

^k^RBD was evaluated using a questionnaire based on the article by [50]

### CSF samples and analysis

CSF samples were obtained by lumbar puncture in the context of the AlphaLewyMA protocol in a diagnostic workup for suspected cognitive decline and underwent a standard protocol (i.e., they were collected in polypropylene tubes [Sarstedt, ref.: 62.610.201] to decrease adsorption of Aβ into the test tubes). Each CSF sample was transported to the laboratory within 4 h after collection; the sample was homogenized on receipt at the laboratory and was then centrifuged at 1700*g* for 10 min at room temperature. All samples were free of blood contamination (the samples were checked visually; if a stain in the sample was detected, the sample was not measured). Samples were then transferred in 0.5-mL polypropylene tubes (Dutscher ref.: 033283) and stored at − 80 °C until analysis. CSF Aβ42, Aβ40, t-Tau, and phospho-tau_181_ were measured by sandwich enzyme-linked immunosorbent assay (ELISA) using commercially available kits (INNOTEST®; Fujirebio Europe, Ghent, Belgium). All assays were performed according to the manufacturer’s instructions, and the methodology did not change during the period in which the analyses were performed. Note that for Aβ1–40, we did not have the same number of patients as for the other biomarkers, either because the dosage was not done systematically or because there was insufficient CSF available to perform an additional Aβ40 assay. For this parameter, 77 patients had a dosage of Aβ40 and were distributed as follows: CS group: *n* = 10, pro-DLB group: *n* = 28, DLB-d group: *n* = 7, pro-AD group: *n* = 17, AD-d group: *n* = 9, pro-AD/DLB group: *n* = 1, and AD/DLB-d group: *n* = 5.

These CSF assays were run as routine clinical neurochemical analyses by technicians trained in CSF analysis at the biochemistry laboratory of University Hospital of Strasbourg. Furthermore, the laboratory participates in the quality control (QC) worldwide program organized by the Alzheimer’s Association QC program for CSF biomarkers. Of note, our results are acceptable in comparison with the other laboratories, thereby further ensuring the quality of the results. Moreover, two internal QC samples per parameter were included in ELISA tests to control for inter-assay variation. Inter-assay coefficients of variations were 2.5–8.7% for Aβ42, 4.4–8.3% for t-Tau, 4.9–16.4% for phospho-Tau_181_, and 1.5–9.0% for Aβ40. The intra-assay variability observed in replicates was less than 10% in all four biomarkers.

The cut-offs used were, therefore, for Aβ42, 500 ng/L (reduced levels were considered pathological); for t-Tau (depending on age), 300 ng/L (< 50 years old), 450 ng/L (50–70 years old), and 500 ng/L (> 70 years old); for phospho-Tau_181_, 60 ng/L; for t-Tau and phospho-Tau_181_, increased levels were considered pathological. For the ratio Aβ42/Aβ40, the cut-off used was 0.05; reduced levels were considered pathological.

CSF levels of total α-syn were assessed using a commercial ELISA for α-syn (hSYN total ELISA; AJ Roboscreen GmbH, Leipzig, Germany) designed and validated for quantification of total α-syn in human CSF [19]. The assay uses a monoclonal capture antibody recognizing amino acids 119 to 126 and a detection antibody to the C-terminus of α-syn. Linearity of the assay is described between 50 and 600 pg/mL. Intra-assay variability of 4.5% was calculated from duplicate analyses and expressed as median of the range to average of the duplicates. Inter-assay imprecision was determined using two quality-control CSF pool samples, low control 10.5% and high control 3.7%.

### Statistical analysis

Statistical analyses were carried out using Graph-Pad PRISM, V.8 (GraphPad, San Diego, CA, USA). Normally distributed data were analyzed using one-way analysis of variance with Tukey’s post hoc analyses to determine between-group differences. In the case of non-Gaussian-distributed parameters, we used the Kruskal-Wallis test with Dunn’s multiple comparison test. In the case of contingency analyses, a *χ*^2^ test was used. Receiver-operating characteristic (ROC) curve analysis was employed to evaluate the diagnostic value of CSF parameters. ROC curve comparisons were performed using MedCalc, V.12.7.0 (MedCalc Software, Ostend, Belgium).

## Results

The study population’s demographic characteristics and mean CSF biomarker values (Aβ42, Aβ40, t-Tau, phospho-Tau_181_, and α-syn) are presented in Table 1. It should be noted that for the comparison of the different parameters studied, the pro-AD/DLB group was excluded from the analyses due to the small number of patients. In summary, for t-Tau, the pro-AD, AD-d, and AD/DLB-d groups had higher values compared to the CS, pro-DLB, and DLB-d groups (see Table 1). For P-Tau, the profile was very similar to that of t-Tau. For Aβ42, there was no significant difference between the CS group and the pro-DLB group but these two groups were significantly different from the pro-AD, AD-d, and AD/DLB-d groups, which all had lower values. However, the DLB-d group was not significantly different from the CS, pro-DLB, pro-AD, AD-d, and AD/DLB-d groups. For Aβ40, there were no differences between the groups. The ratio Aβ42/Aβ40 was not significantly different between the CS, pro-DLB, and DLB-d groups; mean values for the pro-DLB group were significantly higher when compared to each of the AD groups (pro-AD, AD-d, and AD/DLB-d) and those of the CS group were significantly higher compared to the pro-AD and AD/DLB-d groups, whereas those of the DLB-d group were not significantly different from each of the other groups (Table 1).

### α-syn biomarker profile

**Fig. 2:**
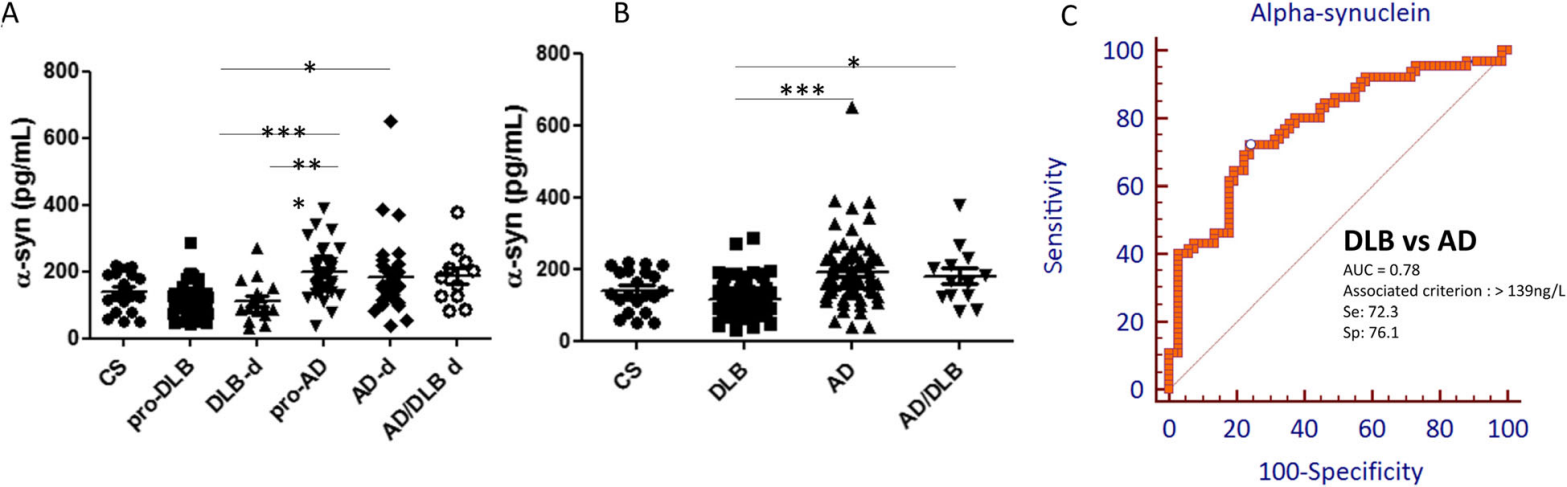
Total alpha-synuclein assay discriminates between AD and DLB (**a**) and **b** scatterplots of CSF alpha-synuclein. **a** CSF concentrations of total alpha-synuclein in each patient group (the number of patients per group was as follows: CS *n* = 21, pro-DLB *n* = 51, DLB-d *n* = 16, pro-AD *n* = 33, AD-d *n* = 32, and AD/DLB-d *n* = 11) and **b** CSF concentration of alpha-synuclein in CS, DLB (pro-DLB + DLB-d), AD (pro-AD + AD-d), and AD/DLB (mixed pathologies: pro-AD/DLB + AD/DLB-d). ***P < 0.001; *P < 0.05. *P* values were calculated using the Kruskal-Wallis test with Dunn’s multiple comparison test. **c** Alpha-synuclein ROC curve between DLB and AD groups. Prodromal and demented patients were pooled in each group. Number of patients per group: DLB *n* = 67, AD *n* = 65*.* Se, sensitivity; Sp, specificity

The results of the α-syn assay are presented in Fig. 2a. No differences were observed between the CS and any of the other groups. α-syn values were similar between the pro-DLB and DLB-d groups and between the pro-AD, AD-d, and AD/DLB-d groups. Interestingly, there was a significant difference between the DLB and AD groups (pro-AD > pro-DLB and DLB-d, *P* < 0.001; AD-d > pro-DLB, *P* < 0.05).

Thus, we observed that the changes in α-syn levels according to pathologies (AD or DLB) appeared from the prodromal stages. For this reason, to discriminate between AD and DLB, the analysis of the diagnostic efficacy of α-syn by the ROC curve, we have pooled the prodromal stages with the demented stages (Fig. 2b, c). The discrimination power of α-syn between the 2 diseases remains moderate (AUC = 0.78, Se = 72.3 and Sp = 76.1 for a 139 ng/L criterion) (Fig. 2b, c and Table 2).

**Table 2:** ROC analysis of CSF parameters for DLB versus AD

CSF variables	Number of patients*	Youden index^**¶**^	Associated criterion^**§**^	Sensitivity (%)	Specificity (%)	AUC (95% CI)
**DLB-(pro+d) vs AD-(pro+d)**						
t-α-synuclein		0.484	> 139 ng/L	72.3	76.1	0.78 (0.70 to 0.85)
t-Tau	DLB *n* = 67 AD *n* = 65	0.713	> 371 ng/L	89.2	82.1	0.92 (0.86 to 0.96)
Phospho-Tau_181_		0.773	> 58 ng/L	90.8	86.6	0.93 (0.87 to 0.97)
Aβ42		0.490	≤ 838 ng/L	90.8	58.2	0.77 (0.69 to 0.84)
t-Tau_phospho-Tau_Aβ42^¥^		0.787	> 0.4714	87.7	91.0	0.95 (0.89 to 0.98)
t-Tau_phospho-Tau_Aβ42_t-α-syn^¥^		0.802	> 0.516	86.2	94.0	0.95 (0.90 to 0.98)
Aβ40	DLB *n* = 34 AD *n* = 25	0.474	> 9563	68.0	79.4	0.70 (0.57 to 0.81)
Aβ42/Aβ40		0.731	≤ 0.0555	76.0	97.1	0.93 (0.83 to 0.98)
t-Tau_phospho-Tau_Aβ42/Aβ40^¥^		0.840	> 0.5368	84.0	100	0.95 (0.86 to 0.99)
t-Tau_phospho-Tau_Aβ42/Aβ40_t-α-syn^¥^		0.840	> 0.5442	84.0	100	0.95 (0.86 to 0.99)
**Pro-DLB vs Pro-AD**						
t-α-synuclein		0.583	> 139 ng/L	81.8	76.5	0.83 (0.73 to 0.90)
t-Tau	DLB *n* = 51 AD *n* = 33	0.701	> 371 ng/L	81.8	88.2	0.89 (0.81 to 0.95)
Phospho-Tau_181_		0.800	> 60 ng/L	87.9	92.2	0.92 (0.84 to 0.97)
Aβ42		0.476	≤ 838 ng/L	84.9	62.8	0.75 (0.64 to 0.84)
t-Tau_phospho-Tau_Aβ42^¥^		0.779	> 0.498	81.8	96.1	0.93 (0.86 to 0.98)
t-Tau_phospho-Tau_Aβ42_t-α-syn^¥^		0.770	> 0.480	84.9	92.2	0.95 (0.88 to 0.98)
Aβ40		0.536	> 9563	75.0	78.6	0.75 (0.60 to 0.87)
Aβ42/Aβ40		0.777	≤ 0.0529	81.3	96.4	0.94 (0.82 to 0.99)
t-Tau_phospho-Tau_Aβ42/Aβ40^¥^	DLB *n* = 28 AD *n* = 16	0.875	> 0.5111	87.5	100	0.95 (0.84 to 0.99)
t-Tau_phospho-Tau_Aβ42/Aβ40_t-α-syn^¥^		0.875	> 0.5148	87.5	100	0.95 (0.83 to 0.99)
**DLB-d vs AD-d**						
t-α-synuclein		0.469	> 92.275 ng/L	90.6	56.3	0.75 (0.60 to 0.86)
t-Tau	DLB *n* = 16 AD *n* = 32	0.750	> 441 ng/L	81.3	93.8	0.94 (0.83 to 0.99)
phospho-Tau_181_		0.750	> 56 ng/L	93.8	81.3	0.93 (0.82 to 0.99)
Aβ42		0.406	≤ 781 ng/L	96.9	43.8	0.73 (0.58 to 0.85)
t-Tau_phospho-Tau_Aβ42^¥^		0.813	> 0.6032	81.3	100	0.96 (0.86 to 1.00)
t-Tau_phospho-Tau_Aβ42_t-α-syn^¥^		0.781	> 0.4877	96.9	81.3	0.96 (0.86 to 1.00)
Aβ40	DLB *n* = 6 AD *n* = 9	0.389	> 9183	55.6	83.3	0.65 (0.37 to 0.87)
Aβ42/Aβ40		0.667	≤ 0.056	66.7	100	0.91 (0.65 to 0.99)
t-Tau_phospho-Tau_Aβ42/Aβ40^¥^		0.778	> 0.5201	77.8	100	0.93 (0.67 to 1.00)
t-Tau_phospho-Tau_Aβ42/Aβ40 t-α-syn^¥^		0.889	> 0.5354	88.9	100	0.96 (0.72 to 1.00)

*Due to missing CSF, some patients could not have an Aβ40 assay

^¶^Youden index: sensitivity + specificity – 1

^**§**^Cut-off associated with the Youden index

^¥^Consideration of three or four parameters with a multiple regression

### Biomarker combinations

Even if the discrimination power of total α-syn seems moderate, it is interesting to determine if, combined with Alzheimer biomarkers, it improves this discrimination power between these two pathologies. As we have previously shown [9, 10], the t-Tau, phospho-Tau, and Aβ42 combination was very effective in discriminating between these two diseases (AUC = 0.95 for DLB-(pro+d) vs AD-(pro+d); Table 2), but unfortunately, the addition of α-syn did not improve this differential diagnosis (AUC = 0.95 for DLB-(pro+d) vs AD-(pro+d); Table 2); the same applies if Aβ42 is replaced by the ratio Aβ42/Aβ40 (t-Tau_phospho-Tau_Aβ42/Aβ40 AUC = 0.95; t-Tau_phospho-Tau_Aβ42/Aβ40_t-α-syn AUC = 0.95 for DLB-(pro+d) vs AD-(pro+d); Table 2).

## Discussion

In summary, the power of α-syn to discriminate between AD and DLB can be considered moderate (Table 2), as previously reported [20, 21]. However, our study shows that the differences observed between AD and DLB appear from the prodromal stage.

Our study has a limitation in that we do not know the exact concentration of hemoglobin in our samples. Indeed, it has been shown that hemoglobin plays a role in α-syn levels in the CSF [22–25]. These studies have shown that beyond 200–500 ng/mL (depending on the study) hemoglobin leads to an artificial increase by interfering with the α-syn assay. However, our samples were visually inspected upon arrival at the laboratory and any samples with pink coloration due to the presence of hemoglobin were rejected. This control is reported to eliminate hemorrhagic samples with more than 500 red cells per μL [26]. Furthermore, on arrival at the laboratory, samples were centrifuged at 1700*g* for 10 min to eliminate as many blood cells as possible that could have contaminated the CSF, thus limiting hemoglobin levels in our samples.

### Early modification of α-syn levels

Regarding the results of the total α-syn assay, we found a significant difference between the DLB group and the AD group. Similar results have previously been highlighted in many publications [20, 21, 26–31], with α-syn levels being higher in AD patients compared to DLB patients. These results have even been confirmed in an autopsy series of patients [32].

The originality of our results is to show that, at the prodromal stage, AD patients had significantly higher α-syn levels than DLB patients. So far, only one recent publication has looked at the prodromal stage and has shown results similar to ours [33]; however, in that study, there were no patients at the demented stage. Thus, we have highlighted more precisely the absence of any change in α-syn levels between the prodromal and dementia stages whatever the pathology (AD or DLB). Thus, total α-syn levels are modified from the prodromal stages (Fig. 2a), suggesting that changes in α-syn levels are implemented early.

### Ability of α-syn to discriminate between neurological controls and DLB and AD patients

α-syn levels of our control subjects were not significantly different from the AD and DLB groups, most likely because of the different neurological pathologies in this group, which made it heterogeneous. In the same way in the literature, it is usually the case that DLB patients were not significantly different from controls [20, 21, 27, 29, 31, 34–38], but a number of publications showed significantly lower levels of α-syn in DLB patients compared to control patients [30, 32, 39, 40]. Garcia-Ayllon et al. even showed that this decrease could take place from the DLB prodromal stage [33].

Interestingly, even if some studies, like ours, showed CSF α-syn levels that were numerically higher, but not significantly so, in AD patients than in CS patients [24], most studies comparing CS patients and AD patients showed that total α-syn levels were significantly higher in AD patients [22, 24, 27, 30, 41], suggesting an α-syn increase in AD patients. On the other hand, by observing the group of patients with AD+DLB comorbidity, it can be seen that the mean α-syn values were at the same level as those of the pure AD groups. This result reinforces the idea that the change in α-syn levels in the CSF is related to an α-syn increase in AD rather than an α-syn decrease in DLB. There are several possible explanations for this increase in AD patients. First, α-syn could be released from damaged neurons [42, 43], as has been hypothesized for the increased levels of CSF tau in AD. Second, an increase in α-syn production was confirmed by Larson et al., who highlighted a 1.67-fold increase in α-syn mRNA levels in the inferior temporal gyrus of AD patients, when compared to age-matched controls, leading to an increase in α-syn monomers even though these AD patients did not have detectable Lewy bodies [44]. Thus, the increase in α-syn production in the brains of AD patients is believed to be responsible for its increase in CSF. In addition, it has been shown that high levels of α-syn may cause cognitive deficits by reducing the release of neurotransmitters by inhibiting the recycling of synaptic vesicles [45]. Thus, it is likely that these increases in soluble α-syn (even in the monomeric form) in the brains of AD patients are the source of an important correlate of decreased cognitive function in AD.

As DLB patients also have neuronal damage, it may seem surprising that there is no α-syn increase in DLB patients. There are two possible explanations for this. First, the aggregating processes of α-syn present in DLB patients are responsible for the decrease in α-syn levels in the CSF, as observed for Aβ42 in AD. The second explanation is that for the same level of cognitive impairment, DLB patients have less neurodegeneration than AD patients [46, 47], which may explain the lower value in DLB patients.

### The different proteinopathies have synergistic adverse effects

Thus, while AD patients have amyloid plaques and DLB patients have Lewy bodies, CSF of AD patients presents an α-syn level increase and CSF of DLB patients an Aβ42 decrease. These results indicate that these pathologies seem to be related in one way or another, which would explain the high frequency of comorbidities, or at least histological hallmarks commonly found between these 2 pathologies. More than 80% of DLB patients showed moderate or abundant cortical amyloid plaques [48], and α-synuclein pathology is also found in up to 50% of patients with AD (for a review, see [49]), suggesting a close link between amyloidopathy and synucleinopathy. In addition, other publications indicate that Tau protein may also have a negative synergy with amyloidopathy and synucleinopathy [50, 51], reinforcing the close link between these different neurodegenerative diseases.

### Ability of the combination of α-syn with standard AD-related biomarkers to discriminate DLB from AD

ROC curves (Table 2) show that even combining α-syn results with Alzheimer biomarkers does not improve the discrimination power compared to the combination of Alzheimer biomarkers alone (t-Tau_phospho-Tau_Aβ42 or Aβ42/Aβ40, AUC = 0.95, Alzheimer biomarkers + α-syn AUC = 0.95). However, this result needs to be put into perspective given that the CSF’s Alzheimer biomarkers are taken into account in the diagnosis, leading to a bias due to an overestimation of the discrimination effectiveness of these Alzheimer biomarkers. Despite taking into account the CSF result, some patients, particularly those clinically considered as Alzheimer’s, present an atypical CSF profile. However, we are quite confident in the diagnosis; in fact, some patients have started to be included in the study from 2013, and consequently, we have a relatively long follow-up of these patients, which has allowed us to reclassify some of them.

## Conclusions

To conclude, the total α-syn assay can participate to discriminate between DLB and AD patients, whatever the stage, but with insufficient specificity and sensitivity. Thus, there is currently a clear lack of new biomarkers specific to DLB for its differential diagnosis. However, other biomarkers are under study. While some are directly related to α-syn, such as the α-syn oligomers, fibrils, or phosphorylation on S129 of α-syn, there are other post-translational modifications or even biomarkers which are unrelated to the direct aggregation processes of α-syn, such as YKL-40, neurogranin, and VILIP-1 (for review, see [8]); yet these biomarkers suffer from a lack of hindsight to determine if they are actually relevant in the biological diagnosis of DLB. Further studies are therefore needed to confirm these results.

## Data Availability

All data generated or analyzed during this study are included in this published article.

## Abbreviations

α-syn: Alpha-synuclein
AD: Alzheimer’s disease
AD-d: AD demented
CS: Control subjects
CSF: Cerebrospinal fluid
DLB: Dementia with Lewy bodies
DLB-d: DLB demented
FCSRT: Free and Cued Selective Reminding Test
MCI: Mild cognitive impairment
MMSE: Mini-Mental State Examination
Phospho-Tau_181_: Tau phosphorylated on residue 181
Pro-AD: Prodromal AD
Pro-DLB: Prodromal DLB
RBD: Rapid eye movement sleep behavior disorder
TMT: Trail Making Test (A or B)
t-Tau: Total Tau
